# MUC1 Regulates Expression of Multiple microRNAs Involved in Pancreatic Tumor Progression, Including the miR-200c/141 Cluster

**DOI:** 10.1371/journal.pone.0073306

**Published:** 2013-10-15

**Authors:** Ashley M. Mohr, Jennifer M. Bailey, Michelle E. Lewallen, Xiang Liu, Prakash Radhakrishnan, Fang Yu, William Tapprich, Michael A. Hollingsworth

**Affiliations:** 1 Eppley Institute for Research in Cancer and Allied Diseases, University of Nebraska Medical Center, Omaha, Nebraska, United States of America; 2 Department of Surgery, the Sol Goldman Pancreatic Cancer Research Center, Johns Hopkins University School of Medicine, Baltimore, Maryland, United States of America; 3 Stowers Institute for Medical Research, Kansas City, Missouri, United States of America; 4 College of Public Health Biostatistics, University of Nebraska Medical Center, Omaha, Nebraska, United States of America; 5 Department of Biology, University of Nebraska at Omaha, Omaha, Nebraska, United States of America; Wayne State University School of Medicine, United States of America

## Abstract

MUC1 is a transmembrane glycoprotein that modulates transcription via its cytoplasmic domain. We evaluated the capacity of MUC1 to regulate the global transcription of microRNAs in pancreatic cancer cells expressing MUC1. Results indicated that MUC1 regulated expression of at least 103 microRNAs. We evaluated further regulation of the microRNA transcript cluster miR-200c/141, which was among the most highly regulated microRNAs. We found that MUC1 directly interacted with ZEB1, a known transcriptional repressor of the miR-200c/141 cluster, at the promoter of miR-200c/141, and further reduced transcript production. These data indicate that signaling through MUC1 influences cancer progression by regulating transcription of microRNAs that are associated with the process of metastasis.

## Introduction

Mucin 1 (MUC1) is present at the apical surface of normal epithelial cells and tumors cells, where it establishes a local extracellular microenvironment and acts as a sensor of the extracellular environment [1]. The carboxyl terminal domain of MUC1 (MUC1.CT) interacts with and is differentially phosphorylated by receptor tyrosine kinases and other serine and threonine kinases [2], which in turn induces its association with transcription factors, translocation to the nucleus and localization at different promoters, where it modulates gene expression [3–7]. Signaling through MUC1 via the C-terminal domain (MUC1.CT) modulates transcriptional programs related to epithelial cell biology [2]. Stimulation of the MET receptor with HGF induces phosphorylation of the tyrosine at YHPM, while stimulation of EGFR induces phosphorylation of the tyrosine at YEKV, and PDGFR phosphorylates tyrosines at YVPP and YHPM [5,6]. Differential phosphorylation allows the MUC1.CT to associate with distinct transcription factors, including β-catenin, p120 catenin, γ-catenin, c-Jun, p53, and ER-α, which, in turn, regulate transcription of multiple genes including MMP1 and CTGF, among others [6,7]. 

 We examined the effects of MUC1 overexpression on global patterns of microRNA (miR) expression in pancreatic tumors, and report here alterations in expression of numerous microRNAs that are associated with cellular processes of tumor progression. MicroRNAs are small regulatory RNAs, 19-23 nucleotides in length, which regulate protein expression by binding to the 3’UTR of mRNA target transcripts. MicroRNAs function by inhibiting translation of the protein (non-homologous binding), or by acting as siRNAs (highly homologous binding), which target mRNAs for degradation. MicroRNAs are differentially expressed in cancer compared to healthy tissue, where they contribute to tumorigenesis. 

 We observed an inverse correlation between miR-200c expression and expression of MUC1, and further evaluated the regulation behind this correlation. MiR-200c regulates a number of proteins associated with epithelial cell differentiation and processes associated with progression of adenocarcinomas [8–11]. Given that MUC1 is known to regulate transcription of a number of processes associated with tumor progression, we hypothesized that transcription of miR-200c was regulated by signal transduction through MUC1. 

Both MUC1 and miR-200c have been associated with tumor progression and epithelial to mesenchymal transition (EMT). EMT is a process in which cells loose cellular contacts, through loss of E-cadherin, thus making them mobile to allow for migration and metastasis. Previous studies have depicted miR-200c as a regulator of EMT by targeting ZEB1, a transcriptional repressor of E-cadherin. ZEB1 also negatively regulates miR-200c; therefore they form a feed-forward loop to stabilize EMT [12]. We report here that miR-200c is regulated by specific phosphorylated forms of MUC1 through direct interaction with ZEB1 at the promoter of miR-200c/141 microRNA cluster.

## Results

### Differential microRNA expression with MUC1

We utilized miRCURY LNA microarrays (Exiqon) to evaluate expression of microRNAs in the context of MUC1 overexpression in pancreatic cancer cells. We examined S2.013 pancreatic cancer cells, with and without overexpression of MUC1, and identified 103 microRNAs altered by MUC1 expression (Table S1). These microRNAs are known and predicted to impact multiple cellular functions that are associated with tumor progression, including proliferation, apoptosis, senescence, metabolism, resistance to chemotherapy, angiogenesis, and EMT (Table S2). The top five up- and down-regulated microRNAs are shown in Table 1. 

**Table 1:** Top 10 differentially expressed microRNAs in S2.013.MUC1 versus S2.013.Neo.

**microRNA**	**Fold Change (log_2_)**
hsa-miR-200c	-8.650552179
hsa-miR-141	-7.231312952
hsa-miR-192	-3.587380659
hsa-miR-33b	-3.502024284
hsa-miR-135b	-3.483740762
hsa-miR-1224-3p	2.378295683
hsa-miR-218	3.137032348
hsa-miR-146a*	3.800029169
hsa-miR-27b*	4.15437337
hsa-miR-130a	4.611699725

### qRT-PCR confirmation of microRNA levels in a subset of microRNAs

**Figure 1:**
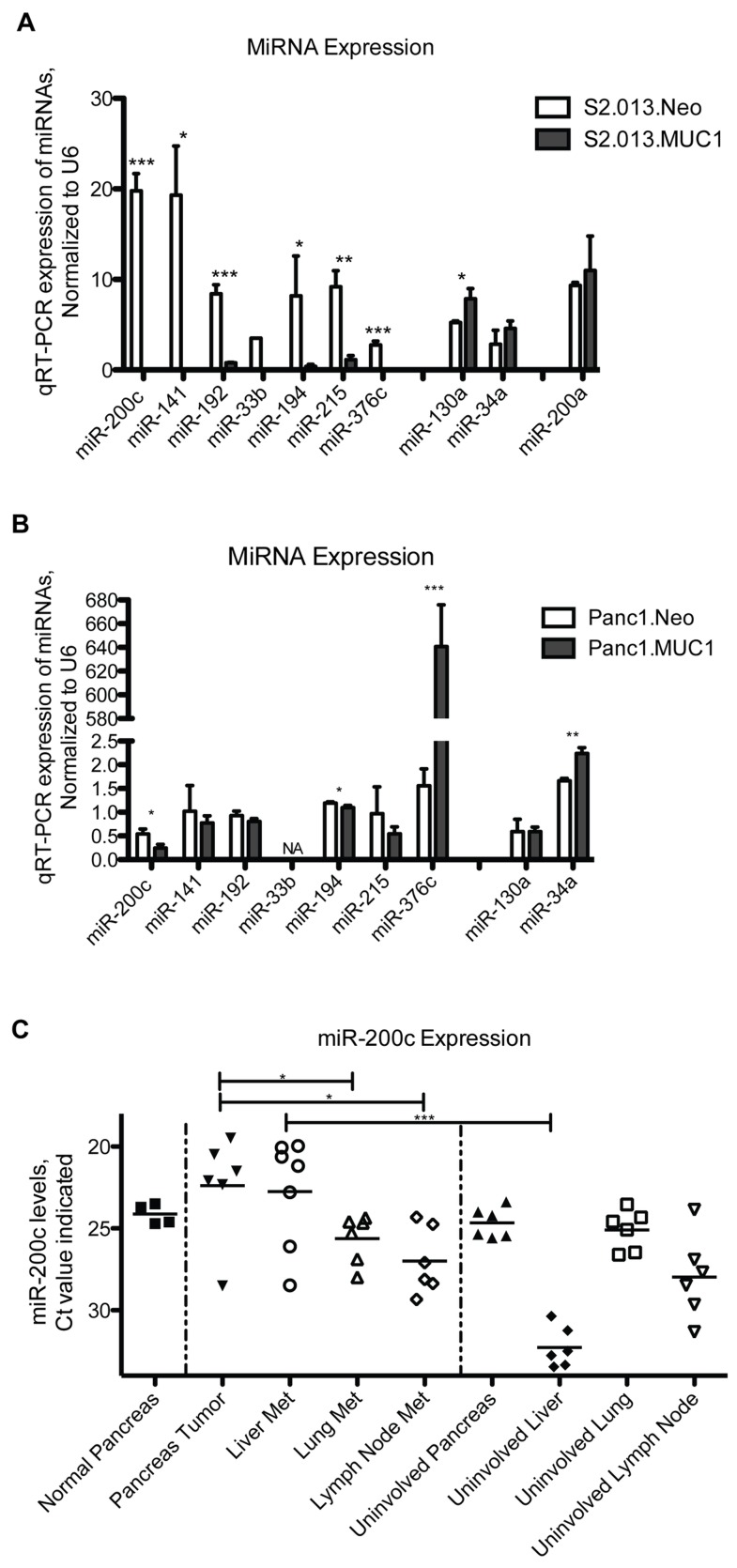
qRT-PCR confirmation of microRNA levels in cells and pancreatic cancer tissue. MicroRNA levels were evaluated in triplicate by qRT-PCR to confirm expression changes identified by microarray analysis (Table 1). MicroRNA levels were normalized to the U6 RNA control. A) MiRs-200c, -141, -192, -33b, -194, -215, and -376c expression levels were evaluated in S2.013.Neo compared to S2.013.MUC1 cells. B) MiRs-200c, -141, -192, -33b, -194, -215, and -376c expression levels were evaluated in Panc1.Neo and Panc1.MUC1 cells. C) Levels of miR-200c were evaluated in a panel of pancreatic cancer tissues. These data are represented by Ct value, where lower Ct values indicate higher levels of miR-200c, and each data point represents a different patient sample. Uninvolved tissue is non-cancerous tissue isolated from a cancer patient. (Student’s t-test: *** p < 0.0005, ** p < 0.005, * p < 0.05).

Quantitative, real time RT-PCR was used to confirm alterations of microRNA expression for a subset of microRNAs that were identified by microarray analysis. We chose to examine: the 4 most down-regulated microRNAs (miR-200c, -141, -192 and -33b); downregulated miR-192 family members (miR-194 and 215, which have been identified as p53-regulated microRNAs); and miR-376c which had been linked to invasion and metastasis. We also evaluated expression of the most highly overexpressed microRNA identified, miR-130a, and another well-known p53-regulated microRNA that was up regulated in the array, miR-34a. MiR-200a was evaluated as a control that was not determined to be differentially expressed on the microRNA microarray, yet related to the miR-200c/141 cluster. We confirmed that levels of the miR-200c/141 transcript were significantly reduced in S2.013.MUC1-overexpressing cells (Figure 1a). Levels of known p53-regulated family members, miRs-192/194/215, were also significantly down regulated in S2.013.MUC1 cells, as well as miR-33b and miR-376c. We confirmed that miR-130a and miR-34a were up-regulated in these cells, although expression of miR-34a did not achieve statistical significance in S2.013.MUC1 cells. Interestingly, levels of another miRNA-200 family member that is separately encoded, miR-200a, were unchanged in S2.013.MUC1 cells. 

We evaluated expression of these microRNAs in a second pancreatic cancer cell line model system of MUC1 overexpression (Panc1) by quantitative RT-PCR. Similar to S2.013.MUC1 cells, miR-200c was down regulated in Panc1.MUC1 cells compared to Panc1.Neo (Figure 1b); however, we did not observe alterations in the levels of miR-141 that is encoded at the same locus. We found that miR-194 was similarly down regulated in the Panc1.MUC1 cells, while miRs-192 and -215 were not altered. Mir-130a was also not significantly altered, but there was an up regulation of miR-34a in Panc1.MUC1 cells. Interestingly, miR-376c showed a dramatic and significant increase in the Panc1.MUC1 cells, whereas it was down regulated in S2.013.MUC1 cells.

### Levels of miR-200c and MUC1 in human pancreatic tumors

We evaluated levels of miR-200c in specimens of primary and metastatic human pancreatic tumors. RNA was isolated from multiple tumors and adjacent uninvolved tissue (tissue that was from the same cancer patient, but lacking tumor) (Figure 1c). These data show that miR-200c levels significantly decrease between primary tumor and metastases to the lung and lymph nodes, but not to the liver. Levels of miR-200c increase in liver metastases, compared to uninvolved liver. These data indicate that expression of miR-200c is maintained but differentially regulated during tumor progression and metastasis.

### MUC1.CT occupancy at the promoter region of miRNA-200c/141

**Figure 2:**
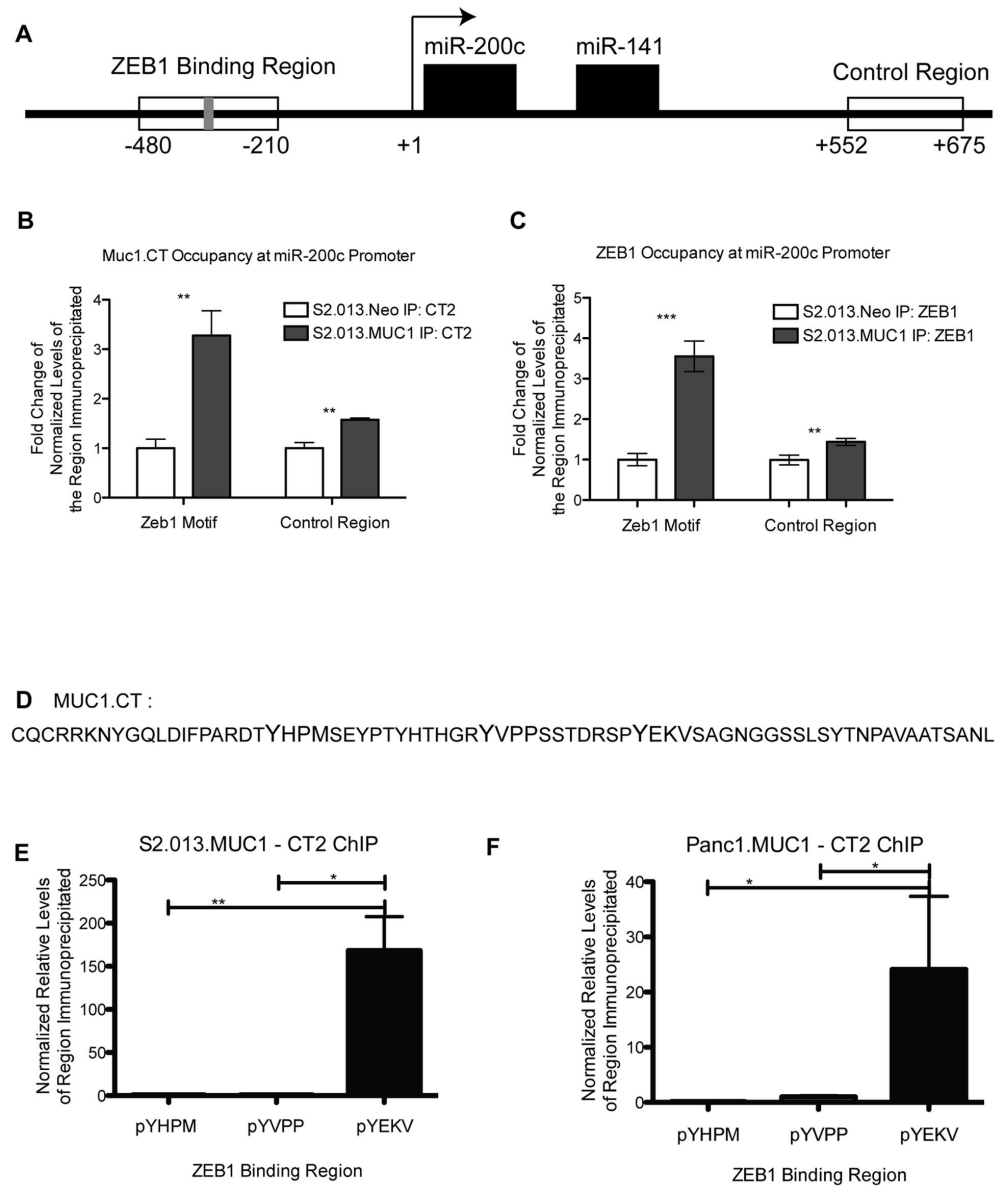
Chromatin Immunoprecipitation confirmation of MUC1.CT and ZEB1 occupancy of the miR-200c/141 promoter. Chromatin immunoprecipitation (ChIP) was utilized to confirm MUC1.CT and ZEB1 occupancy at the miR-200c/141 promoter region. A) Schematic representing the miR-200c/141 promoter region where the ChIP primer sets were designed (-480 to -210, and +552 to +675) and the known ZEB1 binding region (Grey bar). B) ChIP results indicate fold change of normalized relative levels of MUC1.CT occupancy at the ZEB1 binding region (based on qPCR) of the miR-200c/141 promoter in S2.013.MUC1 cells compared to S2.013.Neo cells. C) ChIP results indicate fold change of normalized relative levels of ZEB1 occupancy at the ZEB1 binding region (based on qPCR) of the miR-200c/141 promoter in S2.013.MUC1 cells compared to S2.013.Neo cells. D) Sequence of the MUC1 cytoplasmic tail, indicating three phosphorylated tyrosine motifs that were examined here. E-F) ChIP results indicate relative levels (based on qPCR and normalized to IgG control) of phospho-YHPM, -YVPP, and -YEKV MUC1 cytoplasmic tail at the ZEB1 binding region of miR-200c/141 in the S2.013.MUC1 cells, E, and Panc1.MUC1 cells, F. In both cell lines, only the phospho-YEKV form of MUC1.CT shows significant enrichment at the promoter. All ChIP data was normalized to antibody specific IgG control. (Student’s t-test: *** p < 0.0005, ** p < 0.005, * p< 0.05).

The cytoplasmic tail of MUC1 (MUC1.CT) engages in signal transduction by translocating to the nucleus, where it alters gene expression by associating with and modifying the activity of transcription factors [3–7]. To further investigate the role of MUC1 in regulating the levels of the miR-200c/141 transcript, we utilized chromatin immunoprecipitation (ChIP). ChIP-on-chip data indicated that the MUC1.CT localized to the promoter of miR-200/141 (Figure 2a). The region of the miR-200c/141 promoter bound by the MUC1.CT also contains the known ZEB1 binding domain, a known transcriptional repressor of miR-200c. Figure 2a presents a schematic showing the upstream promoter region of the miR-200c/141 transcript, indicating the ZEB1 binding region (depicted as a grey bar). Primers were designed flanking the promoter region bound by MUC1.CT, as well as a control region (-480 to -210 and +552 to +675, respectively) (Figure 2a). Previous reports have shown that MUC1.CT stabilizes transcription factor occupancy of promoter regions [3,4,6,7,13]. Therefore, we explored in detail the capacity of MUC1.CT to occupy the ZEB1 binding region of the miR-200c/141 locus. ChIP data indicated that MUC1.CT occupied the promoter of miR-200c/141 that contains the ZEB1 binding region (Figure 2b). We therefore examined levels of ZEB1 at the promoter region. These data confirm ZEB1 occupancy at the miR-200c/141 promoter, and show that increased levels of MUC1 correspond to an increase in ZEB1 at the same sites in the promoter by approximately 3.5-fold (Figure 2c).

### Specific phosphorylated form of MUC1.CT localize to the miR-200c promoter

Multiple reports have shown a link between EGFR signaling and E-cadherin (downstream molecule of miR-200c regulation) loss [14–18], therefore, we investigated the contribution of EGFR signaling through MUC1 to the loss of miR-200c. Phosphorylation of MUC1.CT can occur through multiple receptor tyrosine kinases that mediate signaling events, including HGF, MET, PDGF, and EGF [2,5,6]. We evaluated levels of specific phosphorylated forms of MUC1.CT that were localized to the miR-200c/141 promoter. Three antibodies against specific tyrosine-phosphorylated forms of MUC1.CT were used, recognizing the pYHPM, pYVPP, and pYEKV forms of MUC1.CT, which are phosphorylation events that have been shown to be mediated by HGF/c-met, PDGF, and EGF, respectively [5,6,19] (Figure 2d). The pYEKV form of MUC1.CT localized to the ZEB1 binding region of the miR-200c promoter in the S2.013.MUC1 cell line, while the pYHPM and pYVPP forms are not detected at this region (Figure 2e). A second pancreatic cancer cell line overexpressing MUC1, Panc1.MUC1, was investigated for phospho-MUC1.CT occupancy at the miR-200c/141 promoter. We demonstrate that pYEKV MUC1.CT is present at the miR-200c/141 locus in Panc1.MUC1 cells (Figure 2f). These data suggest that EGF phosphorylation of MUC1.CT at YEKV is a principal signaling pathway through which MUC1 regulates levels of miR-200c/141 in this experimental system. 

### MUC1.CT directly interacts with ZEB1 transcription factor

**Figure 3:**
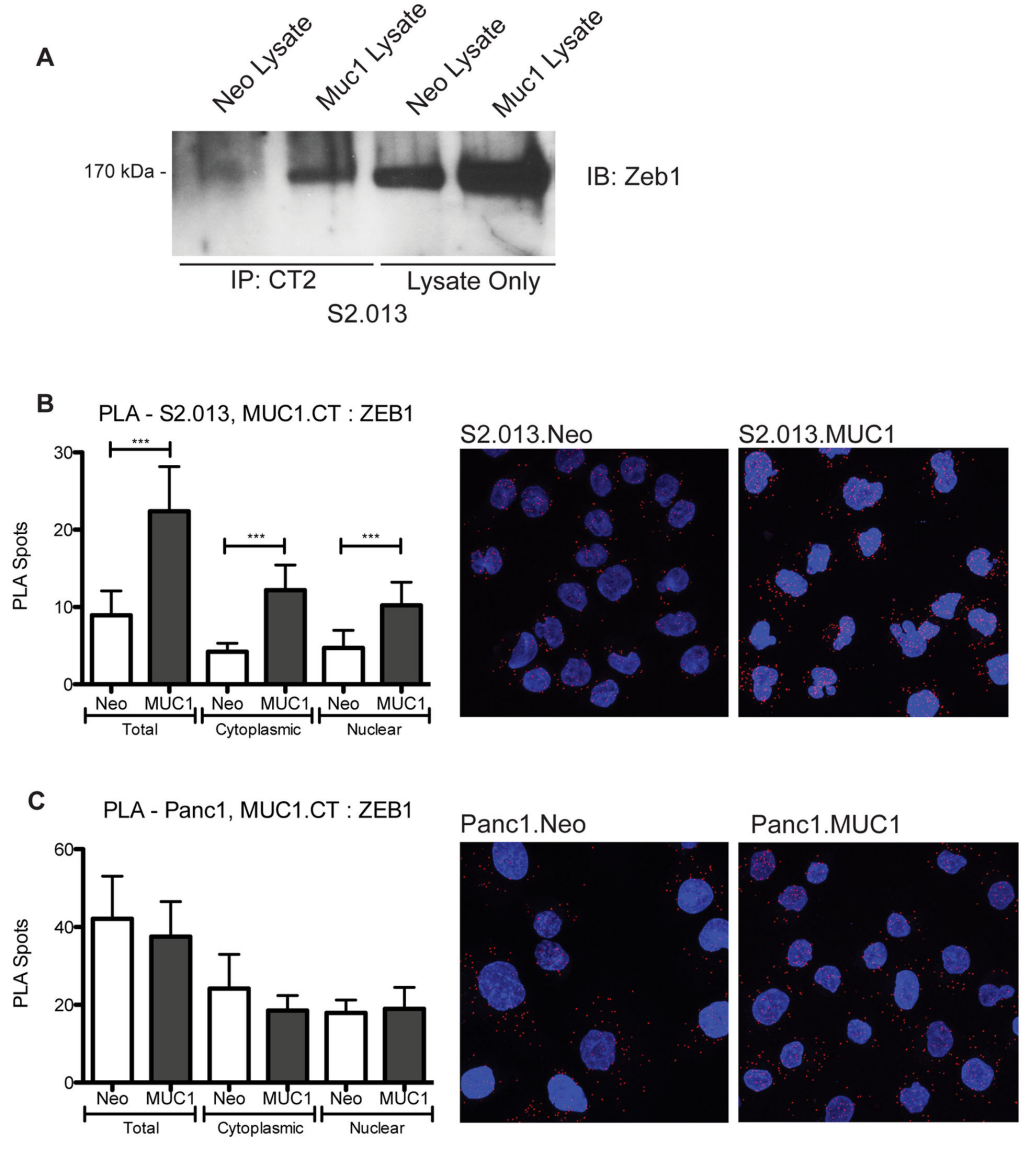
MUC1.CT directly interacts with transcriptional repressor ZEB1. A) Co-immunoprecipitation (Co-IP) was utilized to evaluate the interaction of the MUC1.CT and ZEB1 in S2.013.Neo and S2.013.MUC1 cells. Cell lysates were immunoprecipitated with an antibody against the MUC1.CT, and the subsequent western was blotted with an antibody to ZEB1. Non-immunoprecipitated lysates were used as a loading control, and steady state levels of ZEB1 were higher in S2.013.MUC1 cells compared to S2.013.Neo cells. B-C) Proximity ligation assay (PLA) was used to visualize MUC1.CT and ZEB1 interaction in S2.013.Neo and S2.013.MUC1 cells, B, and Panc1.Neo and Panc1.MUC1 cells, C (with representative compressed z-stack image for each). These data show quantitative levels of MUC1.CT and ZEB1 interactions, indicating that MUC1.CT and ZEB1 interact in both cell lines. In the S2.013.Neo and S2.013.MUC1 cell lines, the interaction was significantly higher in the MUC1 expressing cells, confirming the Co-IP in A. However, there was no significant difference in interaction in the Panc1.Neo and Panc1.MUC1 cells. (Student’s t-test: *** p < 0.0005).

Activated forms of MUC1 directly alter transcription factor occupancy of promoters [2,7]. Therefore, we used co-immunoprecipitation to determine if the MUC1.CT directly interacted with ZEB1. An antibody against the cytoplasmic tail of MUC1 (CT2), and control IgG, were used for immunoprecipitation, and a subsequent immunoblotting with an antibody against ZEB1 showed that MUC1.CT and ZEB1 are present in complexes in S2.013.MUC1 cells (Figure 3a). We also show an increase in steady state ZEB1 levels in S2.013.MUC1 cells, most likely do to the loss of miR-200c and the feed-forward loop that is initiated by down regulation of miR-200c. To further quantify the MUC1.CT interaction with ZEB1, we employed the proximity ligase assay (PLA), which detects in-cell protein-protein interactions as punctate spots that can be quantified and associated with sub-cellular localization. Figure 3b shows quantitative PLA results in the S2.013.Neo and S2.013.MUC1 cell lines as well as a representative compressed z-stack image of the assay. The results of this assay support the co-immunoprecipitation results and demonstrate that MUC1.CT and ZEB1 are colocalized in the cell, at significantly higher levels in the S2.013.MUC1 cells compared to the S2.013.Neo cells. We also evaluated the interaction in Panc1.Neo and Panc1.MUC1 cells and demonstrate that MUC1.CT and ZEB1 interact at similar levels in Panc1.Neo and Panc1.MUC1 cells (Figure 3c). Interestingly, in both the S2.013 and Panc1 cells the interaction occurs in both the nucleus and the cytoplasm. 

### MiR-200c levels in a panel of pancreatic cancer cell lines, primary tumors and liver metastases

**Figure 4:**
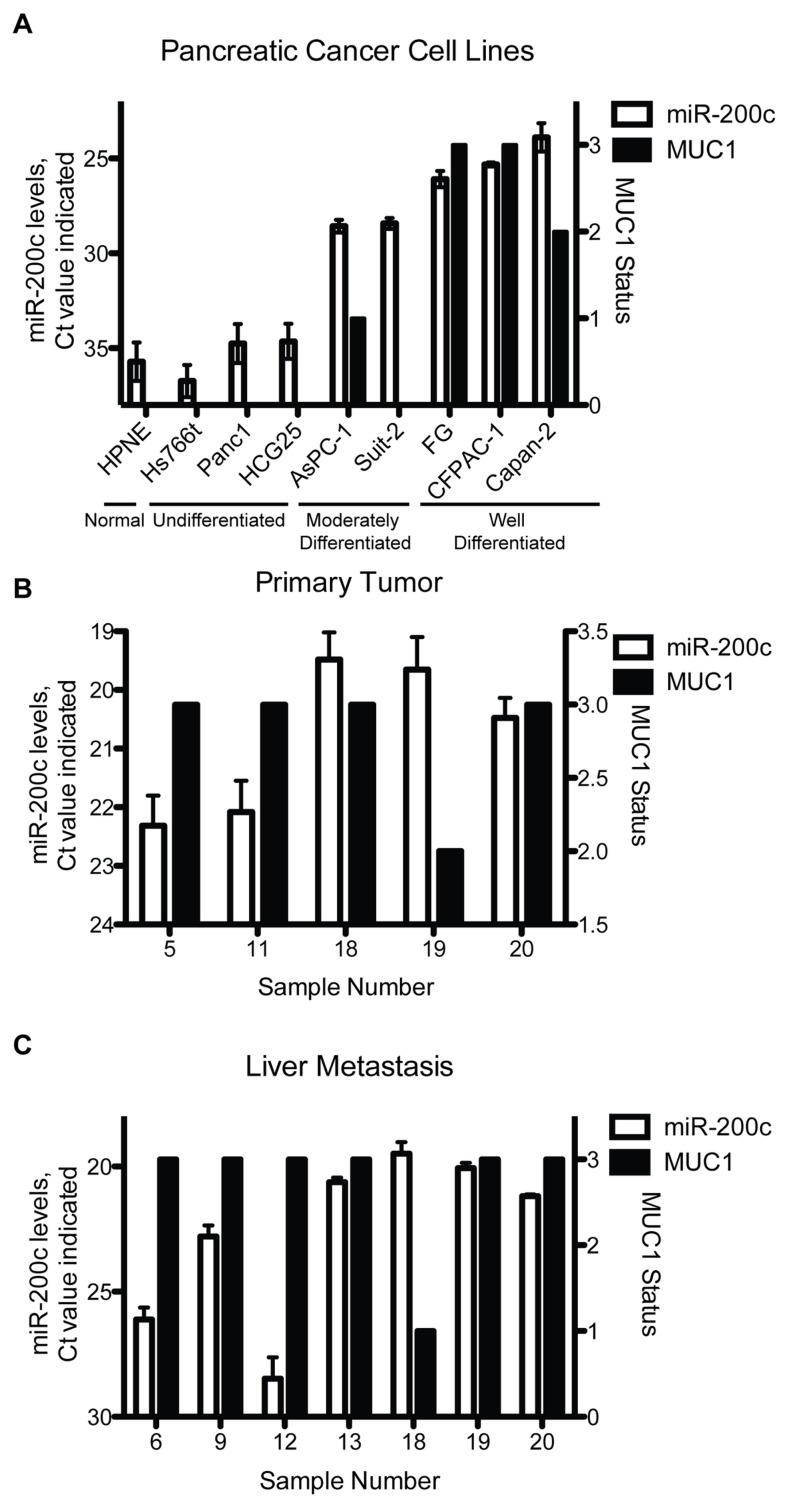
MiR-200c and MUC1 levels in a panel of pancreatic cancer cell lines and tumor samples. Correlation between levels of MUC1 and miR-200c in a panel of pancreatic cancer cell lines and a panel of primary tumor sections and liver metastases. A) MiR-200c levels, indicated by Ct value, are the white bars and correspond to the left y-axis. MUC1 statuses of these cell lines, determined by western blot, are represented in the black bars and right y-axis. Levels of miR-200c and their corresponding MUC1 levels [20], on the left and right y-axis respectively were evaluated in primary pancreatic tissue (B) and liver metastases (C). MUC1 status was determined based on IHC staining from one section per patient; therefore standard deviation was not determined.

To further evaluate miR-200c expression in pancreatic cancer cell lines, we assayed a panel of 8 pancreatic cancer cell lines, as well as a normal, immortalized nestin-expressing pancreatic cell line (HPNE). MiR-200c levels were evaluated in these cells (Figure 4a) and evaluated against MUC1.CT expression (based on western blot, data not shown). MiR-200c levels in these cell lines correlated to differentiation status of the cell lines. Undifferentiated cell lines such as Hs766t, Panc1, and HCG25 have relatively low levels of miR-200c. Moderately differentiated cell lines such as AsPC-1, SUIT-2 and HPAF have moderate levels of miR-200c. Well-differentiated cell lines such as CFPAC-1 and Capan2 have higher levels of miR-200c. We also evaluated levels of MUC1.CT in these cells, depicted with the black bar and using the right y-axis. There was no direct correlation between MUC1 levels and loss of miR-200c in these cell lines. 

We extended this analysis to matched sets of primary pancreatic tumors and metastases. Immunohistochemical staining was previously performed on tissue sections from matched patients and scored on a 1-3 scale, where 1 represents low MUC1 levels, and 3 represents high levels of MUC1 [20]. These matched patient samples from the primary tumor (Figure 4b) or liver metastasis (Figure 4c) are displayed in side by side panels. These data also show there is not a direct correlation between MUC1 expression and miR-200c levels, confirming data found in the pancreatic cancer cell line panel. Thus, taken together, these results demonstrate that MUC1 is not the sole regulator of miR-200c levels but that instead it modulates levels within the context of broader regulatory programs.

### MUC1.CT and ZEB1 interaction in mitotic cells versus non-mitotic cells

**Figure 5:**
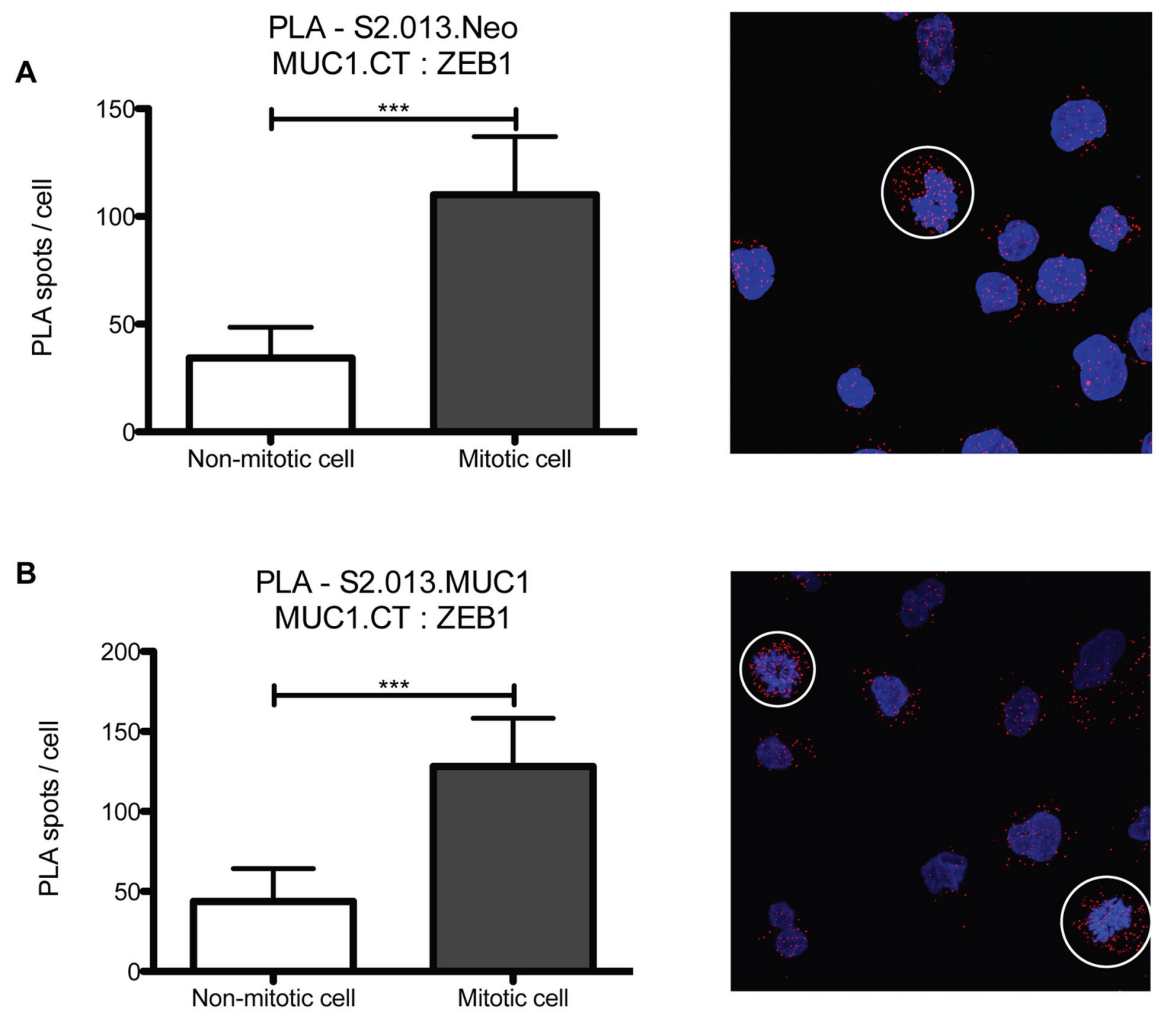
PLA analysis of MUC1.CT interaction with ZEB1 in mitotic cells. Proximity ligation assay of MUC1.CT with ZEB1 was analyzed in mitotic cells versus non-mitotic cells. A) PLA results from mitotic (circled) or non-mitotic cells in S2.013.Neo or S2.013.MUC1, B indicate a roughly 2-3-fold increase in MUC1.CT and ZEB1 interaction. In addition, this interaction occurs mainly in the cytoplasm, not in the nuclei. (*** p < 0.0005).

An incidental finding was noted in our analysis of PLA data in proliferating cells. Cells undergoing mitosis had significantly more MUC1.CT/ZEB1 interactions compared to their non-dividing counterparts (Figures 5a and b). Quantified PLA shows an approximate 2 to 3-fold increase in MUC1.CT:ZEB1 interaction in mitotic cells (circled cells) compared to the non-mitotic cells in each field. 

## Discussion

We demonstrate here that overexpression of MUC1 and concomitant signaling through phosphorylation of the MUC1.CT alters the microRNA profile in pancreatic cancer cells. MicroRNAs that were down regulated by MUC1 overexpression modulate expression of proteins that contribute to the aggressive biological features of pancreatic cancer, including proliferation, apoptosis, senescence, metabolism, resistance to chemotherapy, angiogenesis, and epithelial to mesenchymal transition (Table S2). Both microRNAs of the miR-200c/141 transcript are reported to be down regulated in cancer and to play a role in invasion and metastasis [9,10,21]. MiR-192, and its family members miR-194/215, (known p53-regulated microRNAs) are reported to be down regulated in several cancer studies and may play a role in modulating proliferation and invasion [22–25]. A recent report from Croce et al. identified miRs-200c, -141, -192, -194, and -215 (and other microRNAs) to be upregulated by wild-type p53 [26]. Our study suggests that MUC1.CT alters transcriptional regulation of these miRNAs independent of wildtype p53, as both of the cell lines examined here expressed mutant p53. This suggests that MUC1 interacts with other transcription factors to alter expression miR levels, such as ZEB1 at the miR-200c/141 promoter. MiR-33b regulates fatty acid oxidation and insulin signaling [27], and therefore may play a role in tumor metabolism. MiR-33b was recently characterized in multiple myeloma as a tumor suppressor through targeting PIM-1, an oncogene [28]. MiRNA-130 was found to be up regulated by MUC1, and is increased in non-small cell lung cancer (NSCLC) and associated with metastasis and poor prognosis [29] and chemotherapy resistance to cisplatin [18]. MiR-376 has been implicated in cell survival in ovarian cancer [30]. However, in melanoma miR-376c contributed to progression and metastasis [31]. Interestingly, miR-376c was not consistently regulated between the two cell lines evaluated here, in S2.013.MUC1 cells miR-376 is lost and in Panc1.MUC1 cell line, miR-376c is highly overexpressed. Further evaluation of miR-376c in these cell lines would prove informative in both the regulation of the microRNA (what factors other than MUC1 are playing a role) as well as the functional output (what genes it targets in each cell line). Overall, we show that microRNAs regulated by MUC1 have the potential to influence proliferation, invasion, metastasis, tumor metabolism, and drug resistance.

We previously reported in an abstract and poster presentation that signaling through the MUC1.CT is a potent regulator of miR-200c/141 transcription through interactions with Zeb1 [32]. As we were preparing the final version of this manuscript for submission, an independent report appeared [33] showing results that are consistent with our findings with respect to MUC1 regulation of miR-200c . Our results presented here and those of Rajabi et al [33] present evidence that MUC1.CT binds to the transcriptional repressor ZEB1 and regulates expression of miR-200c. We extend those findings by showing that specific phosphorylated forms of MUC.CT were localized to the ZEB1 binding motif upstream of the miR-200c/141 start site in two independent cell lines. Similar to Rajabi et al, our data suggest that signaling through MUC.CT enables its association with ZEB1 and establishes a feed-forward loop that enhances steady state levels of ZEB1 by repressing miR-200c. We observed that there was an increase in ZEB1 protein levels in S2.013.MUC1 cells (Figure 3a), which is also consistent with the report by Rajabi et al. We also found that an increase in MUC1.CT and ZEB1 interactions in the S2.013 cell line, but not the Panc1 cell line. 

We sought to determine if there were correlations between miR-200c and relative levels of MUC1 expressed by pancreatic cancer cell lines, primary tumors and matched metastatic lesions. Here we report that there was not a correlation between relative levels of MUC1 and miR-200c in a panel of pancreatic cancer cell lines or matched sets of primary tumor and metastases. We interpret these data to suggest that MUC1 is not the sole determining factor that establishes levels of miR-200c, but rather that it modulates levels in the context of broader regulatory pathways. Regulation through MUC1 is dependent on signaling, as evidenced by our pYEKV ChIP results. Thus, this phosphorylated form of MUC1.CT (linked to EGFR signaling) is responsible for establishing a threshold for ZEB1 and MUC1.CT interaction. Overall, we find that the mechanism of MUC1 regulation of the miR-200c/141 cluster is through phosphorylation of the YEKV motif, which promotes an EMT phenotype by regulating miR-200c through interacting with ZEB1. We noted a correlation between miR-200c expression and differentiation status of the pancreatic cancer cell lines tested, in which miR-200c expression was increased in cell lines with higher grades of differentiation. We also note that endogenous MUC1 levels follow a similar pattern.

We also report an incidental finding, in which dividing cells have a 3-fold higher level of MUC1.CT:ZEB1 interactions. Few reports have indicated ZEB1 in conjunction with cellular proliferation. Liu et al showed that in MEFs from ZEB1 mutant mice, cells underwent dose dependent premature senescence [34]. Ahn et al identified that ZEB1 can regulate ∆Np63, a regulator of miR-34a in a mouse model of lung adenocarcinoma. This down regulation of miR-34a is then responsible for pro-metastatic actin cytoskeleton remodeling [35]. These reports, however, focus on ZEB1 as a transcription factor. Most transcription factors are displaced during chromatin condensation in mitosis [36], which accounts for the localization of MUC1.ZEB1 interaction we observe, however it does not account for the increase in interaction. This increase in interaction occurs at the same rate in all dividing cells, without regard to MUC1 levels, which indicates it may be a normal mechanism in dividing cells, and should be further evaluated. It is notable that previous reports have shown that MUC1 can influence cell cycle, and this observation may be related to this effect [37].

In summary, our study provides evidence that MUC1 modulates microRNA levels to aid in cancer progression and metastasis. Signaling through the MUC1 cytoplasmic tail allows for known transcription factor interactions, as well as a novel interaction described here, with ZEB1. We also confirm and extend knowledge of a novel link between MUC1 signaling and EMT, through regulation of the transcription of miR-200c/141.

## Materials and Methods

### Ethics Statement

This research was performed with cell lines that are publically available and with de-identified patient material obtained from deceased patients upon rapid autopsy. Specimens from patients with pancreatic cancer were obtained with written consent and IRB approval IRB 091-01-FB at the University of Nebraska Medical Center through the Rapid Autopsy Program (RAP).

### Cell culture

S2.013 pancreatic cancer cells have previously been described [38], and Panc1 cancer cells were obtained from American Type Culture Collection. Stable expression of MUC1 (flag-epitope tagged) in Panc1 (Panc1.MUC1) and S2.013 (S2.013.MUC1) has previously been described [3] and resulted in robust expression of MUC1 (Figure S1). All cells were grown in Dulbecco’s modified Eagle’s medium supplemented with 10% fetal calf serum, 200 μg/mL G418, and ciprofloxacin. Cells were cultured and maintained in a humidified chamber at 37°C with 5% CO_2_.

### RNA Isolation and Quantitative Real-time PCR

RNA was isolated from S2.013 and Panc1 cells (either MUC1- or Neomycin-expressing) using the *mirVana* miRNA Isolation Kit (Life Technologies, USA) according to manufacturer’s protocol for total RNA extraction. RNA was quantified using a NanoDrop100 spectrophotometer. TaqMan MicroRNA Reverse Transcription Kit (Life Technologies, USA) was used to generate cDNA. TaqMan human MicroRNA assays (Life Technologies, USA) were used to quantify levels of microRNAs and normalized to RNU6b snRNA, according to manufacturer’s protocol, or represented by Ct value. Briefly, 5 ng (S2.013) or 8 ng (Panc1) total RNA was used for the reverse transcription assay and 1.33 μL of this reaction was used for the quantitative real-time PCR assay, in triplicate. 

### RNA isolation from pancreatic cancer tissue

Specimens from patients with pancreatic cancer were obtained with written consent and IRB approval IRB 091-01 at the University of Nebraska Medical Center through the Rapid Autopsy Program (RAP). Specimens were obtained within 3 hours post-mortem to ensure minimal RNA degradation. Samples were either flash frozen in liquid nitrogen or placed directly in formalin fixative for paraffin embedding. Frozen tissues from primary pancreatic tumors, liver, lung and lymph node metastases, as well as uninvolved tissue from these sites were utilized for RNA isolation. We also obtained normal pancreas samples from non-cancer patients from the UNMC Tissue Sciences Facility. Frozen specimens were placed in liquid nitrogen and ground to a fine powder with a sterilized mortar and pestle (without thawing), and immediately placed in the *mirVana* microRNA lysis buffer. RNA isolation was then completed according to manufacturers protocol as stated above. 

### MicroRNA microarray

MiRCURY LNA microarrays (Exiqon, Denmark) were used according to manufacturer protocol. Briefly, RNA isolated from S2.013.Neo and S2.013.MUC1 cells was labeled with either Hy3^TM^ or Hy5^TM^, hybridized to the array slide, and subsequently washed. The arrays were then scanned and analyzed using GenePix Pro 6 (Molecular Devices, USA). The array files were read in and preprocessed by background correction, within-array normalization through Loess normalization. The normalized data was used to obtain values for the differential expression between samples. For each cell line, microRNAs with average intensity values lower than the 5^th^ percentile average intensity values of the arrays were filtered out. MicroRNAs with at least 2-fold change were designated as differentially expressed. These data were deposited in Gene Expression Omnibus, Accession number GSE48185.

### Co-immunoprecipitation

Nuclear lysates were prepared by lysing cells using a hypotonic/hypertonic lysis method. Cells were scraped into a hypotonic buffer (10 mM Hepes pH 7.9, 10 mM KCl, and 0.1 mM EDTA) to lyse the cells, while leaving the nuclei intact. Centrifugation at 6,000 x *g* for 2 minutes was used to collect the nuclei. The supernatant was kept for the cytosolic fraction and the pelleted nuclei were washed twice with hypotonic buffer. Hypertonic lysis buffer (20 mM Hepes pH 7.9, 0.4 M NaCL, and 1 mM EDTA) was added to lyse the nuclei. This fraction was centrifuged at 16,000 x *g* for 5 minutes to remove cell debris from the nuclear lysates. These buffers were supplemented with 0.1 mM PMSF and complete protease inhibitor tablet (Roche Diagnostics, USA). Co-immunoprecipitations were performed with 2 μg CT2 antibody and an isotype-matched IgG as control.

### Chromatin Immunoprecipitation and Real-time PCR analysis

Chromatin immunoprecipitations were performed according to a protocol adapted from Affymetrix. Briefly, 5.0x10^7^ cells were washed in 1XPBS and incubated in 5mM dimethyl 3,3’-dithiobispropioimidate (DTBP, Pierce Biotechnology) for 30 minutes at 4°C. Cells were rinsed and incubated in 1% HPLC-grade formaldehyde (Sigma) for 10 minutes at room temperature. Crosslinking reactions were quenched with 2.5M glycine for 5 minutes. Cells were lysed in ChIP Lysis Buffer (10mM Tris-HCl pH 7.5, 10mM NaCl, 3mM MgCl2, 0.5% IGEPAL, 1mM PMSF). Nuclei were collected and sonicated in Pre-IP Dilution Buffer (10mM Tris-HCl pH 7.5, 10mM NaCl, 3mM MgCl2, 1mM CaCl2, 4% IGEPAL, 1mM PMSF) at duty cycle 60% and amplitude 50% with 1-minute pulses followed by 1 minute rests. Equal amounts of chromatin were immunoprecipitated with specific antibodies as indicated or IgG isotype control in the presence of protease and phosphatase inhibitors. Chromatin was diluted in 5 volumes of IP Dilution Buffer (20mM Tris-HCl, 2mM EDTA, 1% Triton X-100, 150 mM NaCl) with protease and phosphatase inhibitors. 10 ug antibody was added to chromatin and incubated rotating overnight at 4°C. 200 ul Protein G Sepharose bead slurry (Sigma) was added to immune complexes and incubated for 3 hours with rotation at room temperature. Bead-antibody-antigen complexes were washed with successive washes of ChIP Wash 1 (20mM Tris-HCl pH 8.0, 2mM EDTA, 1% Triton X-100, 150 mM NaCl), ChIP Wash 2 (20mM Tris-HCl pH 8.0, 2mM EDTA, 1% Triton X-100, 0.1% SDS, 500 mM NaCl) and ChIP Wash 3 (10mM Tris-HCl pH 8.0, 1mM EDTA, 0.25M LiCl, 0.5% IGEPAL, 0.5% deoxycholate) with protease and phosphatase inhibitors. Immunoprecipitates were eluted in Elution Buffer (25mM Tris-HCl pH 7.5, 10mM EDTA, 0.5%SDS) and decrosslinked with 100mM DTT and 1 ug/ul Proteinase K at 65°C overnight. Decrosslinked chromatin was purified using Qiaquick PCR Purification Kit (Qiagen) and subjected to real time PCR analysis. 3.0 uL chromatin was prepared in Sybr green master mix (Applied Biosystems) and subjected to quantitative real-time PCR analysis using ABI 7500 thermocycler. Each reaction was repeated in triplicate and the experiments were repeated at least twice to confirm reproducibility. Values were obtained for the threshold cycle (Ct) for each gene and data were analyzed using the standard curve method. Values were normalized to enrichment of a genomic region within a housekeeping gene (β-glucuronidase, GUSB). GUSB validated primer pair was purchased from SABiosciences. 

Values are reported as enrichment relative to IgG ChIP, +/- SEM. Oligonucleotide sequences for detection of miR-200c regulatory regions are presented in Table S3. GUSB validated primer pair was purchased from SABiosciences. 

### Proximity Ligation Assay

Reagents for the proximity ligation assay (PLA) were purchased from Olink (Sweden). PLA provides evidence for protein-protein interactions directly in cells by detecting their proximity. Primary antibodies against two proteins of interest generated in different species are added to the cells. Secondary antibodies (probes) are conjugated to complimentary DNA sequences, that when in close proximity (>40nm) anneal to form circular DNA. Amplification of this circular DNA template with flourescent probes results in punctate spots for each protein-protein interaction. PLA was preformed according to manufacturer protocol. Briefly, cells were seeded overnight on 12 mm poly-L-lysine coated coverslips in a 12-well plate. The next day cells were washed twice with PBS, fixed with 4% paraformaldehyde with 120 mM sucrose for 15 minutes, and 0.1 M glycine was added for 15 minutes. Cells were permeabilized for 15 minutes with 0.15% TritonX-100 and 1% BSA in PBS. Cells were washed again twice with PBS. Duolink blocking solution was added to the cells and incubated at 37°C for 30 minutes in a humidity chamber. Blocking solution was removed and primary antibody, diluted in antibody diluent, was applied to the cells overnight at 4°C in a humidity chamber. Cells were washed three times in PLA wash buffer A prior to PLA probe addition for one hour at 37°C in a humidity chamber. Cells were washed twice with PLA wash buffer A prior to ligase addition for thirty minutes at 37°C in a humidity chamber. Cells were washed again twice before addition of polymerase solution for 100 minutes at 37°C in a humidity chamber. Cells were washed twice in PLA wash buffer B and once in 0.01X PLA wash buffer B. Coverslips were then mounted in Vectashield with Dapi and sealed with clear nail polish. Confocal microscopy was performed on a Zeiss 710 Confocal Laser Scanning Microscope at 63x. Z-stacks were captured, and BlobFinder (Sweden) software was used to analyze PLA data.

### Western blot and scoring of MUC1.CT in pancreatic cancer cell lines

Protein lysates were isolated from pancreatic cancer cell lines by using a whole cell RIPA lysis buffer (150 mM NaCl, 1.0% IGEPAL, 0.5% sodium deoxycholate, 0.1% SDS, 50 mM Tris, pH, 8.0). Westerns were run with approximately 50 µg total protein on 10% Bis-Tris gel (Life Technologies, USA), and transferred to a nylon membrane. Membranes were blocked in 5% non-fat dried milk with 1% TBST, and incubated with CT2 antibody (1:500) for 3 hours, and secondary antibody for 1 hour at room temperature. Levels of MUC1.CT were designated a number value from 0-3 depending on their levels.

### Statistical analysis

Statistical analysis was performed using GraphPad Prism statistical software. A two-tailed student’s *t* test was used to determine significance. p < 0.05 was considered significant.

## Supporting Information

Figure S1 **MUC1 overexpression in S2.013 and Panc1 cells.**
(PDF)

Table S1 **MicroRNAs differentially expressed in S2.013.MUC1 cells versus S2.013.Neo cells.**
(DOCX)

Table S2 **Functional characterization of miRNAs differentially regulated by MUC1 expression.**
(DOCX)

Table S3 **Oligonucleotide sequences for detection of 200C regulatory regions.**
(PDF)
