# Supplementary material for: MUC1 Regulates Expression of Multiple microRNAs Involved in Pancreatic Tumor Progression, Including the miR-200c/141 Cluster
Source: PLoS One. 2013 Oct 15;8(10):e73306. doi: 10.1371/journal.pone.0073306 (PMC3797065; doi:10.1371/journal.pone.0073306)
Supplement: Figure S1 — MUC1 overexpression in S2.013 and Panc1 cells. (PDF) [file pone.0073306.s001.pdf]

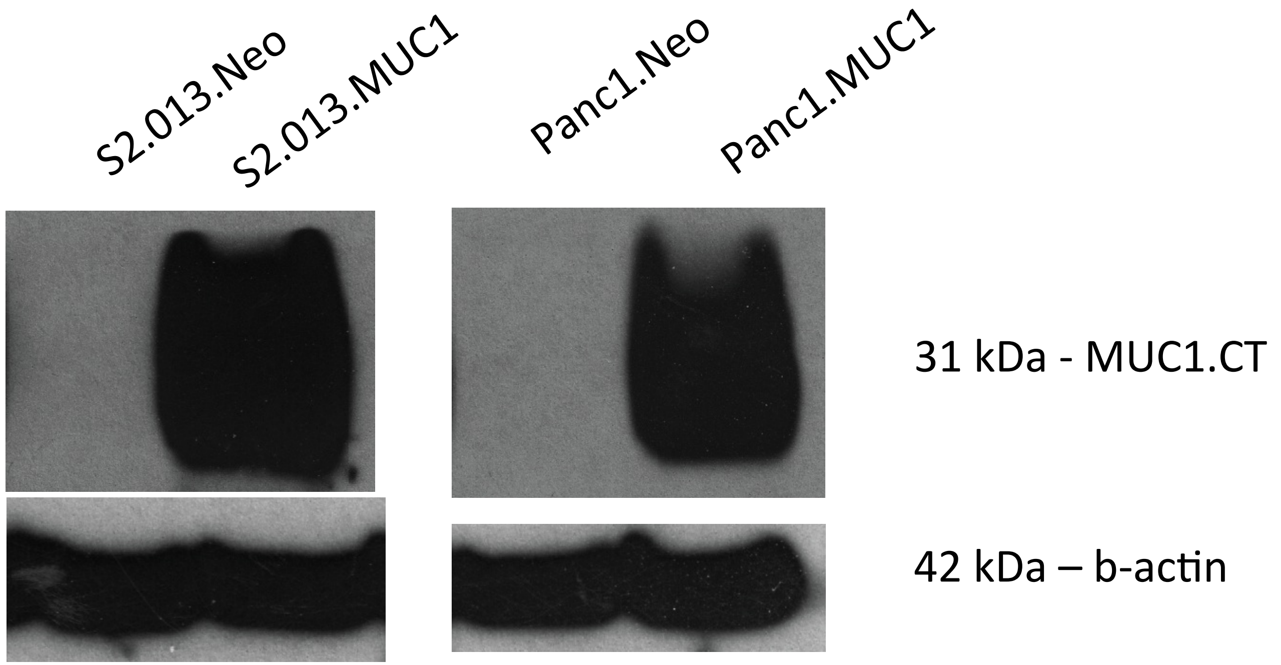

Figure S1. MUC1.CT overexpression in S2.013 and Panc1 cells. Western blot of S2.013.Neo, S2.013.MUC1, Panc1.Neo and Panc1.MUC1 showing the level of MUC1 overexpression. B-actin was used as a loading control.
