# Supplementary material for: MUC1 Regulates Expression of Multiple microRNAs Involved in Pancreatic Tumor Progression, Including the miR-200c/141 Cluster
Source: PLoS One. 2013 Oct 15;8(10):e73306. doi: 10.1371/journal.pone.0073306 (PMC3797065; doi:10.1371/journal.pone.0073306)
Supplement: Table S1 — MicroRNAs differentially expressed in S2.013.MUC1 cells versus S2.013.Neo cells. (DOCX) [file pone.0073306.s002.docx]

Table S1. MicroRNAs differentially expressed in S2.013.MUC1 cells versus S2.013.Neo cells.

| MicroRNA | Fold Change (log scale) |
| --- | --- |
| hsa-miR-200c | -8.650552179 |
| hsa-miR-141 | -7.231312952 |
| hsa-miR-192 | -3.587380659 |
| hsa-miR-33b | -3.502024284 |
| hsa-miR-135b | -3.483740762 |
| hsa-miR-942 | -3.422325926 |
| hsa-miR-220a | -3.409465443 |
| hsa-miR-194 | -3.379898109 |
| hsa-miR-16-1* | -3.216267408 |
| hsa-miR-922 | -3.19781709 |
| hsa-miR-215 | -2.797075516 |
| hsa-miR-326 | -2.758127539 |
| hsa-miR-136 | -2.39919442 |
| hsa-miR-432 | -2.229924198 |
| hsa-miR-376c | -2.05603923 |
| hsa-miR-192* | -1.92488932 |
| hsa-miR-875-5p | -1.859937296 |
| hsa-miR-30b* | -1.845302173 |
| hsa-miR-122* | -1.817867831 |
| hsa-miR-377 | -1.802459787 |
| hsa-miR-654-3p | -1.782422751 |
| hsa-miR-1249 | -1.767746499 |
| hsa-miR-490-5p | -1.750073061 |
| hsa-miR-30c-2* | -1.728845083 |
| hsa-miR-625 | -1.593366994 |
| hsa-miR-135a | -1.558648026 |
| hsa-miR-155 | -1.533626111 |
| hsa-miR-1237 | -1.530147002 |
| hsa-miR-100 | -1.526150425 |
| hsa-let-7d* | -1.509398035 |
| hsa-miR-1226 | -1.503023296 |
| hsa-miR-760 | -1.401287809 |
| hsa-miR-224 | -1.392692059 |
| hsa-miR-1287 | -1.37060278 |
| hsa-miR-649 | -1.324818733 |
| hsa-miR-1200 | -1.298021231 |
| hsa-miR-147b | -1.276868163 |
| hsa-miR-125b | -1.236097448 |
| hsa-miR-142-3p | -1.206063999 |
| hsa-miR-376a | -1.156810972 |
| hsa-miR-361-3p | -1.149770145 |
| hsa-miR-484 | -1.122481164 |
| hsa-miR-19b | -1.091000076 |
| hsa-miR-584 | -1.076737936 |
| hsa-miR-495 | -1.076481313 |
| hsa-miR-1279 | -1.056937296 |
| hsa-miR-503 | -1.039791353 |
| hsa-miR-629 | -1.024266004 |
| hsa-miR-320c | -1.021023891 |
| hsa-miR-19b-2* | -1.006942528 |
| hsa-miR-622 | -1.001834416 |
| hsa-miR-10b | 1.009578678 |
| hsa-miR-891b | 1.024373134 |
| hsa-miR-372 | 1.03456438 |
| hsa-miR-202 | 1.066293603 |
| hsa-miR-146a | 1.076315307 |
| hsa-miR-146b-5p | 1.081366292 |
| hsa-miR-27b | 1.088603128 |
| hsa-miR-214* | 1.100395501 |
| hsa-miR-1293 | 1.109769987 |
| hsa-miR-1225-3p | 1.145240679 |
| hsa-miR-34a | 1.163195501 |
| hsa-miR-766 | 1.190659697 |
| hsa-miR-940 | 1.200417994 |
| hsa-miR-421 | 1.213160616 |
| hsa-miR-140-5p | 1.216730981 |
| hsa-miR-635 | 1.228943162 |
| hsa-miR-587 | 1.232672276 |
| hsa-miR-9 | 1.234002595 |
| hsa-miR-23b | 1.249379446 |
| hsa-miR-1253 | 1.268132533 |
| hsa-miR-511 | 1.2762097 |
| hsa-miR-342-5p | 1.277779941 |
| hsa-miR-181c* | 1.30306102 |
| hsa-miR-662 | 1.321912731 |
| hsa-miR-623 | 1.336598539 |
| hsa-miR-656 | 1.352619872 |
| hsa-miR-1229 | 1.359708093 |
| hsa-miR-558 | 1.404955967 |
| hsa-miR-181d | 1.412262396 |
| hsa-miR-577 | 1.453247866 |
| hsa-miR-140-3p | 1.465191542 |
| hsa-miR-380* | 1.496537311 |
| hsa-miR-1184 | 1.52063031 |
| hsa-miR-519b-3p | 1.558304234 |
| hsa-miR-513a-3p | 1.560306319 |
| hsa-miR-33a* | 1.613985994 |
| hsa-miR-362-5p | 1.747804566 |
| hsa-miR-451 | 1.747804566 |
| hsa-miR-501-3p | 1.747804566 |
| hsa-miR-1256 | 1.781721058 |
| hsa-miR-1179 | 2.006781857 |
| hsa-miR-616 | 2.03736956 |
| hsa-miR-1205 | 2.093443688 |
| hsa-miR-496 | 2.135720449 |
| hsa-miR-1178 | 2.135720449 |
| hsa-miR-518d-3p | 2.258528129 |
| hsa-miR-532-5p | 2.362704448 |
| hsa-miR-1224-3p | 2.378295683 |
| hsa-miR-218 | 3.137032348 |
| hsa-miR-146a* | 3.800029169 |
| hsa-miR-27b* | 4.15437337 |
| hsa-miR-130a | 4.611699725 |

Microarray data showing miRNAs differentially expressed between S2.013.MUC1 and S2.013.Neo cells. Refer to Methods section for more information.
