# Supplementary material for: MUC1 Regulates Expression of Multiple microRNAs Involved in Pancreatic Tumor Progression, Including the miR-200c/141 Cluster
Source: PLoS One. 2013 Oct 15;8(10):e73306. doi: 10.1371/journal.pone.0073306 (PMC3797065; doi:10.1371/journal.pone.0073306)
Supplement: Table S2 — Functional characterization of miRNAs differentially regulated by MUC1 expression. (DOCX) [file pone.0073306.s003.docx]

Table S2. Functional characterization of miRNAs differentially regulated by MUC1 expression.

| Characterization | miRNAs | Regulation by MUC1 | References |
| --- | --- | --- | --- |
| Cellular Proliferation/  Apoptosis/  Senescence | miR-192, miR-194, miR-16-1*(3p), miR-215, miR-136, miR-376c, miR-30c-2*(3p), miR-135a, miR-155, miR-100, miR-1226, miR-760, miR-224, miR-376a, miR-19b, miR-495, miR-503, miR-622, miR-135b, miR-33b, miR-125b | Down | [1,2,3,4,5,6,7,8,9,10,11,12,13,14,15,16,17,18,19,20,21,22,23,24,25,26,27,28,29,30] |
|  | miR-372, miR-146a, miR-146b-5p, miR-34a, miR-421, miR-140-5p, miR-9, miR-511, miR-181d, miR-380*(5p), miR-519-3p, miR-451, miR-616, miR-532-5p, miR-218, miR-27b | Up | [31,32,33,34,35,36,37,38,39,40,41,42,43,44,45,46,47,48,49,50,51] |
| Cellular Metabolism | miR-33b, miR-326 | Down | [52,53] |
|  | miR-33a*(3p), miR-23b | Up | [54,55] |
| Cellular Migration/  Invasion | miR-200c, miR-141, miR-192, miR-215, miR-376c, miR-326, miR-625-5p, miR-135a, miR-155, miR-224, miR-125b, miR-376a, miR-19b, miR-584, miR-495, miR-503, miR-622, miR-135b, miR-33b | Down | [4,7,16,18,23,24,25,27,29,30,56,57,58,59,60,61,62,63,64,65,66,67] |
|  | miR-10b, miR-146a, miR-146b-5p, miR-27b, miR-34a, miR-421, miR-9, miR-23b, miR-451, miR-218 | Up | [32,36,41,47,50,68,69,70,71,72,73,74,75,76,77,78,79,80,81] |
| EMT | miR-200c, miR-141, miR-194, miR-495 | Down | [62,65,82] |
|  | miR-372, miR-34a | Up | [83,84] |
| Chemotherapy Resistance | miR-141, miR-215, miR-326, miR-376c, miR-30c-2*(3p), miR-155, miR-19b, miR-503 | Down | [10,12,85,86,87,88,89,90] |
|  | miR-513a-3p, miR-451, miR-130a | Up | [46,91,92,93] |
| Angiogenesis | miR-361-3p, miR-135b, miR-125b | Down | [94,95,96,97] |
|  | miR-27b, miR-9, miR-130a | Up | [74,75,98] |
| Unknown Biological Function in Cancer | miR-942, miR-922, miR-432, miR-192*(3p), miR-875-5p, miR-30b*(3p), miR-122*(3p), miR-377, miR-654-3p, miR-1249, miR-490-5p, miR-1237, let-7d*(3p), miR-1287, miR-649, miR-320c, miR-19b-2*(5p) | Down |  |
|  | miR-891b, miR-202, miR-241*(5p), miR-1293, miR-1225-3p, miR-766, miR-940, miR-635, miR-587, miR-1253, miR-342-5p, miR-181c*(3p), miR-623, miR-656, miR-1229, miR-558, miR-577, miR-501-3p, miR-1256, miR-1179, miR-1205, miR-496, miR-1178, miR-518d-3p, miR-1224-3p, miR-146a*(3p), miR-27b*(5p) | Up |  |

Characterization based on previously published data (references appended). For miRNAs that were annotated with * in the original data, (5p) or (3p) was added to correct for current nomenclature.

1. Braun CJ, Zhang X, Savelyeva I, Wolff S, Moll UM, et al. (2008) p53-Responsive micrornas 192 and 215 are capable of inducing cell cycle arrest. Cancer Res 68: 10094-10104.

2. Feng S, Cong S, Zhang X, Bao X, Wang W, et al. MicroRNA-192 targeting retinoblastoma 1 inhibits cell proliferation and induces cell apoptosis in lung cancer cells. Nucleic Acids Res 39: 6669-6678.

3. Georges SA, Biery MC, Kim SY, Schelter JM, Guo J, et al. (2008) Coordinated regulation of cell cycle transcripts by p53-Inducible microRNAs, miR-192 and miR-215. Cancer Res 68: 10105-10112.

4. Jin Z, Selaru FM, Cheng Y, Kan T, Agarwal R, et al. MicroRNA-192 and -215 are upregulated in human gastric cancer in vivo and suppress ALCAM expression in vitro. Oncogene 30: 1577-1585.

5. Song B, Wang Y, Kudo K, Gavin EJ, Xi Y, et al. (2008) miR-192 Regulates dihydrofolate reductase and cellular proliferation through the p53-microRNA circuit. Clin Cancer Res 14: 8080-8086.

6. Chiang Y, Zhou X, Wang Z, Song Y, Liu Z, et al. Expression levels of microRNA-192 and -215 in gastric carcinoma. Pathol Oncol Res 18: 585-591.

7. Pichiorri F, Suh SS, Rocci A, De Luca L, Taccioli C, et al. Downregulation of p53-inducible microRNAs 192, 194, and 215 impairs the p53/MDM2 autoregulatory loop in multiple myeloma development. Cancer Cell 18: 367-381.

8. Yang Y, Wu J, Guan H, Cai J, Fang L, et al. MiR-136 promotes apoptosis of glioma cells by targeting AEG-1 and Bcl-2. FEBS Lett 586: 3608-3612.

9. Meng HT, Zhu L, Ni WM, You LS, Jin J, et al. Triptolide inhibits the proliferation of cells from lymphocytic leukemic cell lines in association with downregulation of NF-kappaB activity and miR-16-1*. Acta Pharmacol Sin 32: 503-511.

10. Ye G, Fu G, Cui S, Zhao S, Bernaudo S, et al. MicroRNA 376c enhances ovarian cancer cell survival by targeting activin receptor-like kinase 7: implications for chemoresistance. J Cell Sci 124: 359-368.

11. Fu G, Ye G, Nadeem L, Ji L, Manchanda T, et al. MicroRNA-376c Impairs Transforming Growth Factor-beta and Nodal Signaling to Promote Trophoblast Cell Proliferation and Invasion. Hypertension 61: 864-872.

12. Jia W, Eneh JO, Ratnaparkhe S, Altman MK, Murph MM MicroRNA-30c-2* expressed in ovarian cancer cells suppresses growth factor-induced cellular proliferation and downregulates the oncogene BCL9. Mol Cancer Res 9: 1732-1745.

13. Wu H, Huang M, Cao P, Wang T, Shu Y, et al. MiR-135a targets JAK2 and inhibits gastric cancer cell proliferation. Cancer Biol Ther 13: 281-288.

14. Wu S, Lin Y, Xu D, Chen J, Shu M, et al. MiR-135a functions as a selective killer of malignant glioma. Oncogene 31: 3866-3874.

15. Yamada Y, Hidaka H, Seki N, Yoshino H, Yamasaki T, et al. Tumor-suppressive microRNA-135a inhibits cancer cell proliferation by targeting the c-MYC oncogene in renal cell carcinoma. Cancer Sci 104: 304-312.

16. Zhou W, Li X, Liu F, Xiao Z, He M, et al. MiR-135a promotes growth and invasion of colorectal cancer via metastasis suppressor 1 in vitro. Acta Biochim Biophys Sin (Shanghai) 44: 838-846.

17. Ali S, Banerjee S, Logna F, Bao B, Philip PA, et al. Inactivation of Ink4a/Arf leads to deregulated expression of miRNAs in K-Ras transgenic mouse model of pancreatic cancer. J Cell Physiol 227: 3373-3380.

18. Giangreco AA, Vaishnav A, Wagner D, Finelli A, Fleshner N, et al. Tumor Suppressor microRNAs, miR-100 and -125b, are Regulated by 1,25-dihydroxyvitamin D in Primary Prostate Cells and in Patient Tissue. Cancer Prev Res (Phila).

19. Liu J, Lu KH, Liu ZL, Sun M, De W, et al. MicroRNA-100 is a potential molecular marker of non-small cell lung cancer and functions as a tumor suppressor by targeting polo-like kinase 1. BMC Cancer 12: 519.

20. Jin C, Rajabi H, Kufe D miR-1226 targets expression of the mucin 1 oncoprotein and induces cell death. Int J Oncol 37: 61-69.

21. Kim SY, Lee YH, Bae YS MiR-186, miR-216b, miR-337-3p, and miR-760 cooperatively induce cellular senescence by targeting alpha subunit of protein kinase CKII in human colorectal cancer cells. Biochem Biophys Res Commun 429: 173-179.

22. Wang Y, Lee AT, Ma JZ, Wang J, Ren J, et al. (2008) Profiling microRNA expression in hepatocellular carcinoma reveals microRNA-224 up-regulation and apoptosis inhibitor-5 as a microRNA-224-specific target. J Biol Chem 283: 13205-13215.

23. Zhang Y, Takahashi S, Tasaka A, Yoshima T, Ochi H, et al. Involvement of microRNA-224 in cell proliferation, migration, invasion, and anti-apoptosis in hepatocellular carcinoma. J Gastroenterol Hepatol 28: 565-575.

24. Zehavi L, Avraham R, Barzilai A, Bar-Ilan D, Navon R, et al. Silencing of a large microRNA cluster on human chromosome 14q32 in melanoma: biological effects of mir-376a and mir-376c on insulin growth factor 1 receptor. Mol Cancer 11: 44.

25. Xu XM, Wang XB, Chen MM, Liu T, Li YX, et al. MicroRNA-19a and -19b regulate cervical carcinoma cell proliferation and invasion by targeting CUL5. Cancer Lett 322: 148-158.

26. Jiang X, Huang H, Li Z, He C, Li Y, et al. MiR-495 is a tumor-suppressor microRNA down-regulated in MLL-rearranged leukemia. Proc Natl Acad Sci U S A 109: 19397-19402.

27. Zhou J, Wang W Analysis of microRNA expression profiling identifies microRNA-503 regulates metastatic function in hepatocellular cancer cell. J Surg Oncol 104: 278-283.

28. Han Z, Yang Q, Liu B, Wu J, Li Y, et al. MicroRNA-622 functions as a tumor suppressor by targeting K-Ras and enhancing the anticarcinogenic effect of resveratrol. Carcinogenesis 33: 131-139.

29. Zhang L, Sun ZJ, Bian Y, Kulkarni AB MicroRNA-135b acts as a tumor promoter by targeting the hypoxia-inducible factor pathway in genetically defined mouse model of head and neck squamous cell carcinoma. Cancer Lett 331: 230-238.

30. Tian Z, Zhao JJ, Tai YT, Amin SB, Hu Y, et al. Investigational agent MLN9708/2238 targets tumor suppressor microRNA-33b in MM cells. Blood.

31. Voorhoeve PM, le Sage C, Schrier M, Gillis AJ, Stoop H, et al. (2007) A genetic screen implicates miRNA-372 and miRNA-373 as oncogenes in testicular germ cell tumors. Adv Exp Med Biol 604: 17-46.

32. Chen G, Umelo IA, Lv S, Teugels E, Fostier K, et al. miR-146a Inhibits Cell Growth, Cell Migration and Induces Apoptosis in Non-Small Cell Lung Cancer Cells. PLoS One 8: e60317.

33. Geraldo MV, Yamashita AS, Kimura ET MicroRNA miR-146b-5p regulates signal transduction of TGF-beta by repressing SMAD4 in thyroid cancer. Oncogene 31: 1910-1922.

34. Chang TC, Wentzel EA, Kent OA, Ramachandran K, Mullendore M, et al. (2007) Transactivation of miR-34a by p53 broadly influences gene expression and promotes apoptosis. Mol Cell 26: 745-752.

35. Chen F, Hu SJ Effect of microRNA-34a in cell cycle, differentiation, and apoptosis: a review. J Biochem Mol Toxicol 26: 79-86.

36. Zhang Y, Gong W, Dai S, Huang G, Shen X, et al. Downregulation of human farnesoid X receptor by miR-421 promotes proliferation and migration of hepatocellular carcinoma cells. Mol Cancer Res 10: 516-522.

37. Hao J, Zhang S, Zhou Y, Liu C, Hu X, et al. MicroRNA 421 suppresses DPC4/Smad4 in pancreatic cancer. Biochem Biophys Res Commun 406: 552-557.

38. Zhang Y, Eades G, Yao Y, Li Q, Zhou Q Estrogen receptor alpha signaling regulates breast tumor-initiating cells by down-regulating miR-140 which targets the transcription factor SOX2. J Biol Chem 287: 41514-41522.

39. Cekaite L, Rantala JK, Bruun J, Guriby M, Agesen TH, et al. MiR-9, -31, and -182 deregulation promote proliferation and tumor cell survival in colon cancer. Neoplasia 14: 868-879.

40. Selcuklu SD, Donoghue MT, Rehmet K, de Souza Gomes M, Fort A, et al. MicroRNA-9 inhibition of cell proliferation and identification of novel miR-9 targets by transcriptome profiling in breast cancer cells. J Biol Chem 287: 29516-29528.

41. Zheng L, Qi T, Yang D, Qi M, Li D, et al. microRNA-9 suppresses the proliferation, invasion and metastasis of gastric cancer cells through targeting cyclin D1 and Ets1. PLoS One 8: e55719.

42. Zhang C, Chi YL, Wang PY, Wang YQ, Zhang YX, et al. miR-511 and miR-1297 inhibit human lung adenocarcinoma cell proliferation by targeting oncogene TRIB2. PLoS One 7: e46090.

43. Wang XF, Shi ZM, Wang XR, Cao L, Wang YY, et al. MiR-181d acts as a tumor suppressor in glioma by targeting K-ras and Bcl-2. J Cancer Res Clin Oncol 138: 573-584.

44. Swarbrick A, Woods SL, Shaw A, Balakrishnan A, Phua Y, et al. miR-380-5p represses p53 to control cellular survival and is associated with poor outcome in MYCN-amplified neuroblastoma. Nat Med 16: 1134-1140.

45. Abdelmohsen K, Srikantan S, Kuwano Y, Gorospe M (2008) miR-519 reduces cell proliferation by lowering RNA-binding protein HuR levels. Proc Natl Acad Sci U S A 105: 20297-20302.

46. Bandres E, Bitarte N, Arias F, Agorreta J, Fortes P, et al. (2009) microRNA-451 Regulates Macrophage Migration Inhibitory Factor Production and Proliferation of Gastrointestinal Cancer Cells. Clin Cancer Res 15: 2281-2290.

47. Godlewski J, Bronisz A, Nowicki MO, Chiocca EA, Lawler S microRNA-451: A conditional switch controlling glioma cell proliferation and migration. Cell Cycle 9: 2742-2748.

48. Ma S, Chan YP, Kwan PS, Lee TK, Yan M, et al. MicroRNA-616 induces androgen-independent growth of prostate cancer cells by suppressing expression of tissue factor pathway inhibitor TFPI-2. Cancer Res 71: 583-592.

49. Kitago M, Martinez SR, Nakamura T, Sim MS, Hoon DS (2009) Regulation of RUNX3 tumor suppressor gene expression in cutaneous melanoma. Clin Cancer Res 15: 2988-2994.

50. Venkataraman S, Birks DK, Balakrishnan I, Alimova I, Harris PS, et al. MicroRNA 218 acts as a tumor suppressor by targeting multiple cancer phenotype-associated genes in medulloblastoma. J Biol Chem 288: 1918-1928.

51. Lee JJ, Drakaki A, Iliopoulos D, Struhl K MiR-27b targets PPARgamma to inhibit growth, tumor progression and the inflammatory response in neuroblastoma cells. Oncogene 31: 3818-3825.

52. Davalos A, Goedeke L, Smibert P, Ramirez CM, Warrier NP, et al. miR-33a/b contribute to the regulation of fatty acid metabolism and insulin signaling. Proc Natl Acad Sci U S A 108: 9232-9237.

53. Kefas B, Comeau L, Erdle N, Montgomery E, Amos S, et al. Pyruvate kinase M2 is a target of the tumor-suppressive microRNA-326 and regulates the survival of glioma cells. Neuro Oncol 12: 1102-1112.

54. Goedeke L, Vales-Lara FM, Fenstermaker M, Cirera-Salinas D, Chamorro-Jorganes A, et al. A regulatory role for miRNA-33* in controlling lipid metabolism gene expression. Mol Cell Biol.

55. Gao P, Tchernyshyov I, Chang TC, Lee YS, Kita K, et al. (2009) c-Myc suppression of miR-23a/b enhances mitochondrial glutaminase expression and glutamine metabolism. Nature.

56. White NM, Khella HW, Grigull J, Adzovic S, Youssef YM, et al. miRNA profiling in metastatic renal cell carcinoma reveals a tumour-suppressor effect for miR-215. Br J Cancer 105: 1741-1749.

57. Chen Y, Zhang J, Wang H, Zhao J, Xu C, et al. miRNA-135a promotes breast cancer cell migration and invasion by targeting HOXA10. BMC Cancer 12: 111.

58. Liu S, Guo W, Shi J, Li N, Yu X, et al. MicroRNA-135a contributes to the development of portal vein tumor thrombus by promoting metastasis in hepatocellular carcinoma. J Hepatol 56: 389-396.

59. Zhang GJ, Xiao HX, Tian HP, Liu ZL, Xia SS, et al. Upregulation of microRNA-155 promotes the migration and invasion of colorectal cancer cells through the regulation of claudin-1 expression. Int J Mol Med 31: 1375-1380.

60. Huang L, Dai T, Lin X, Zhao X, Chen X, et al. MicroRNA-224 targets RKIP to control cell invasion and expression of metastasis genes in human breast cancer cells. Biochem Biophys Res Commun 425: 127-133.

61. Li Z, Cao Y, Jie Z, Liu Y, Li Y, et al. miR-495 and miR-551a inhibit the migration and invasion of human gastric cancer cells by directly interacting with PRL-3. Cancer Lett 323: 41-47.

62. Hwang-Verslues WW, Chang PH, Wei PC, Yang CY, Huang CK, et al. miR-495 is upregulated by E12/E47 in breast cancer stem cells, and promotes oncogenesis and hypoxia resistance via downregulation of E-cadherin and REDD1. Oncogene 30: 2463-2474.

63. Guo XB, Jing CQ, Li LP, Zhang L, Shi YL, et al. Down-regulation of miR-622 in gastric cancer promotes cellular invasion and tumor metastasis by targeting ING1 gene. World J Gastroenterol 17: 1895-1902.

64. Valencia K, Martin-Fernandez M, Zandueta C, Ormazabal C, Martinez-Canarias S, et al. miR-326 associates with biochemical markers of bone turnover in lung cancer bone metastasis. Bone 52: 532-539.

65. Burk U, Schubert J, Wellner U, Schmalhofer O, Vincan E, et al. (2008) A reciprocal repression between ZEB1 and members of the miR-200 family promotes EMT and invasion in cancer cells. EMBO Rep 9: 582-589.

66. Wang M, Li C, Nie H, Lv X, Qu Y, et al. Down-regulated miR-625 suppresses invasion and metastasis of gastric cancer by targeting ILK. FEBS Lett 586: 2382-2388.

67. Ueno K, Hirata H, Shahryari V, Chen Y, Zaman MS, et al. Tumour suppressor microRNA-584 directly targets oncogene Rock-1 and decreases invasion ability in human clear cell renal cell carcinoma. Br J Cancer 104: 308-315.

68. Yao Q, Cao Z, Tu C, Zhao Y, Liu H, et al. MicroRNA-146a acts as a metastasis suppressor in gastric cancer by targeting WASF2. Cancer Lett.

69. Hwang SJ, Seol HJ, Park YM, Kim KH, Gorospe M, et al. MicroRNA-146a suppresses metastatic activity in brain metastasis. Mol Cells 34: 329-334.

70. Li Y, Vandenboom TG, 2nd, Wang Z, Kong D, Ali S, et al. miR-146a suppresses invasion of pancreatic cancer cells. Cancer Res 70: 1486-1495.

71. Katakowski M, Zheng X, Jiang F, Rogers T, Szalad A, et al. MiR-146b-5p suppresses EGFR expression and reduces in vitro migration and invasion of glioma. Cancer Invest 28: 1024-1030.

72. Xia H, Qi Y, Ng SS, Chen X, Li D, et al. (2009) microRNA-146b inhibits glioma cell migration and invasion by targeting MMPs. Brain Res 1269: 158-165.

73. Ahn YH, Gibbons DL, Chakravarti D, Creighton CJ, Rizvi ZH, et al. ZEB1 drives prometastatic actin cytoskeletal remodeling by downregulating miR-34a expression. J Clin Invest 122: 3170-3183.

74. Zhang H, Qi M, Li S, Qi T, Mei H, et al. microRNA-9 targets matrix metalloproteinase 14 to inhibit invasion, metastasis, and angiogenesis of neuroblastoma cells. Mol Cancer Ther 11: 1454-1466.

75. Zhuang G, Wu X, Jiang Z, Kasman I, Yao J, et al. Tumour-secreted miR-9 promotes endothelial cell migration and angiogenesis by activating the JAK-STAT pathway. EMBO J 31: 3513-3523.

76. Liu Y, Yan W, Zhang W, Chen L, You G, et al. MiR-218 reverses high invasiveness of glioblastoma cells by targeting the oncogenic transcription factor LEF1. Oncol Rep 28: 1013-1021.

77. Ishteiwy RA, Ward TM, Dykxhoorn DM, Burnstein KL The microRNA -23b/-27b cluster suppresses the metastatic phenotype of castration-resistant prostate cancer cells. PLoS One 7: e52106.

78. Wang Y, Rathinam R, Walch A, Alahari SK (2009) Suppression of tumorigenicity 14 (ST14) gene is a target for miR-27b, and the inhibitory effect of ST14 on cell growth is independent of miR-27b regulation. J Biol Chem.

79. Liu Z, Zhu J, Cao H, Ren H, Fang X miR-10b promotes cell invasion through RhoC-AKT signaling pathway by targeting HOXD10 in gastric cancer. Int J Oncol 40: 1553-1560.

80. Negrini M, Calin GA (2008) Breast cancer metastasis: a microRNA story. Breast Cancer Res 10: 203.

81. Loftus JC, Ross JT, Paquette KM, Paulino VM, Nasser S, et al. miRNA expression profiling in migrating glioblastoma cells: regulation of cell migration and invasion by miR-23b via targeting of Pyk2. PLoS One 7: e39818.

82. Dong P, Kaneuchi M, Watari H, Hamada J, Sudo S, et al. MicroRNA-194 inhibits epithelial to mesenchymal transition of endometrial cancer cells by targeting oncogene BMI-1. Mol Cancer 10: 99.

83. Subramanyam D, Lamouille S, Judson RL, Liu JY, Bucay N, et al. Multiple targets of miR-302 and miR-372 promote reprogramming of human fibroblasts to induced pluripotent stem cells. Nat Biotechnol 29: 443-448.

84. Du R, Sun W, Xia L, Zhao A, Yu Y, et al. Hypoxia-induced down-regulation of microRNA-34a promotes EMT by targeting the Notch signaling pathway in tubular epithelial cells. PLoS One 7: e30771.

85. Song B, Wang Y, Titmus MA, Botchkina G, Formentini A, et al. Molecular mechanism of chemoresistance by miR-215 in osteosarcoma and colon cancer cells. Mol Cancer 9: 96.

86. Kong W, He L, Coppola M, Guo J, Esposito NN, et al. MicroRNA-155 regulates cell survival, growth, and chemosensitivity by targeting FOXO3a in breast cancer. J Biol Chem 285: 17869-17879.

87. Kurokawa K, Tanahashi T, Iima T, Yamamoto Y, Akaike Y, et al. Role of miR-19b and its target mRNAs in 5-fluorouracil resistance in colon cancer cells. J Gastroenterol 47: 883-895.

88. Park YT, Jeong JY, Lee MJ, Kim KI, Kim TH, et al. MicroRNAs overexpressed in ovarian ALDH1-positive cells are associated with chemoresistance. J Ovarian Res 6: 18.

89. Liang Z, Wu H, Xia J, Li Y, Zhang Y, et al. Involvement of miR-326 in chemotherapy resistance of breast cancer through modulating expression of multidrug resistance-associated protein 1. Biochem Pharmacol 79: 817-824.

90. van Jaarsveld MT, Helleman J, Boersma AW, van Kuijk PF, van Ijcken WF, et al. miR-141 regulates KEAP1 and modulates cisplatin sensitivity in ovarian cancer cells. Oncogene.

91. Bian HB, Pan X, Yang JS, Wang ZX, De W Upregulation of microRNA-451 increases cisplatin sensitivity of non-small cell lung cancer cell line (A549). J Exp Clin Cancer Res 30: 20.

92. Zhang X, Zhu J, Xing R, Tie Y, Fu H, et al. miR-513a-3p sensitizes human lung adenocarcinoma cells to chemotherapy by targeting GSTP1. Lung Cancer 77: 488-494.

93. Yang LY, Wang HJ, Jia XB, Wang X, Luo J, et al. [Expression of miR-130a in cisplatin resistant cell lines of ovarian cancer]. Sichuan Da Xue Xue Bao Yi Xue Ban 43: 60-64.

94. Matsuyama H, Suzuki HI, Nishimori H, Noguchi M, Yao T, et al. miR-135b mediates NPM-ALK-driven oncogenicity and renders IL-17-producing immunophenotype to anaplastic large cell lymphoma. Blood 118: 6881-6892.

95. He J, Jing Y, Li W, Qian X, Xu Q, et al. Roles and Mechanism of miR-199a and miR-125b in Tumor Angiogenesis. PLoS One 8: e56647.

96. Dal Monte M, Landi D, Martini D, Bagnoli P Antiangiogenic role of miR-361 in human umbilical vein endothelial cells: functional interaction with the peptide somatostatin. Naunyn Schmiedebergs Arch Pharmacol 386: 15-27.

97. Kanitz A, Imig J, Dziunycz PJ, Primorac A, Galgano A, et al. The expression levels of microRNA-361-5p and its target VEGFA are inversely correlated in human cutaneous squamous cell carcinoma. PLoS One 7: e49568.

98. Kuehbacher A, Urbich C, Zeiher AM, Dimmeler S (2007) Role of Dicer and Drosha for endothelial microRNA expression and angiogenesis. Circ Res 101: 59-68.
