# Supplementary material for: MUC1 Regulates Expression of Multiple microRNAs Involved in Pancreatic Tumor Progression, Including the miR-200c/141 Cluster
Source: PLoS One. 2013 Oct 15;8(10):e73306. doi: 10.1371/journal.pone.0073306 (PMC3797065; doi:10.1371/journal.pone.0073306)
Supplement: Table S3 — Oligonucleotide sequences for detection of 200C regulatory regions. (PDF) [file pone.0073306.s004.pdf]

Supplementary Table 3

Oligonucleotide sequences for detection of 200C regulatory regions.

Region -410/-280 (ZEB1 Binding Region)	
Forward:	CTTAAAGCCCCTTCGTCTCC
Reverse:	GATTTACCCACCCTCATCCC
Region +552/+675 (Control Region)	
Forward:	CTGACCTTCACCCCTCTGGA
Reverse:	GGAGGTGCCTAGGGAACCAG
